# Correlation of Mid-Trimester Spot Urinary Albumin: Creatinine Ratio With the Adverse Pregnancy Outcome

**DOI:** 10.7759/cureus.36186

**Published:** 2023-03-15

**Authors:** Bharti Singh, K Pushpalatha, Shweta Patel

**Affiliations:** 1 Obstetrics & Gynaecology, All India Institute of Medical Sciences, Bhopal, Bhopal, IND

**Keywords:** neonatal outcome, endothelial dysfunction, pregnancy outcome, microalbuminuria, urinary albumin: creatinine ratio

## Abstract

Background

Urinary albumin creatinine ratio (ACR) is a known method of measurement of microalbuminuria. Microalbuminuria may be an early marker of endothelial dysfunction which can lead to various complications during the course of pregnancy. The objective of our study was to evaluate the correlation of mid-trimester spot urinary ACR with the pregnancy outcome.

Material and methods

We performed a prospective cohort study in the department of Obstetrics & Gynaecology, All India Institute of Medical Sciences, Bhopal, for a period of one year. We studied 130 antenatal women between 14 and 28 weeks of gestation after obtaining written informed consent. The patients with ongoing urinary tract infection (UTI), pre-existing hypertension, or diabetes were excluded. Urinary samples were examined for spot ACR, and the women were followed until delivery. Primary maternal outcomes were development of gestational hypertension, pre-eclampsia, gestational diabetes mellitus (GDM), and preterm labour. Neonatal outcome was assessed in terms of birth weight, the APGAR (Appearance, Pulse, Grimace, Activity, Respiration) scores, and neonatal intensive care unit (NICU) admission.

Results

In our study, mean urinary ACR was 19.07±12.94 mcg/mg and median urinary ACR (IQR) was 18 (9.43-25.25) mcg/mg. Prevalence of microalbuminuria in our study was 19.2%. It was observed that urinary ACR level was significantly higher in women with maternal complications like GDM, gestational hypertension, preeclampsia, and preterm labour. Also, mean urinary ACR of women who developed preeclampsia was higher (37.53±31.85) compared to women who developed gestational hypertension (27.40±9.71). Urinary ACR level was significantly higher in babies with low APGAR scores and in babies who needed NICU admission (p value < 0.05). The sensitivity and specificity of spot urinary ACR to predict GDM and preeclampsia were found to be good as calculated from the receiver operating curve.

Conclusion

We found definite correlation of higher values of mid-trimester urinary ACR with the adverse pregnancy outcome.

## Introduction

Microalbuminuria is defined as an abnormal increase in albumin excretion rate within the specific range of 30-299 milligrams of albumin/gram of creatinine [1]. Spot urinary albumin: creatinine ratio (ACR) can be unique as it has the ability to detect the small amount of albumin in the urine, which can preclude the requirement of 24 hour urinary protein measurement [2]. The National Kidney Foundation, the American Diabetes Association, and the National Institutes of Health advocate the estimation of albumin in the urine by the method of albumin: creatinine ratio [3]. Evidence from the studies suggest that microalbuminuria detected in the general population is associated with obesity, diabetes, overt renal disease, and hypertension and there is future risk of cardiovascular complications and premature death [4,5]. The risk of complications can increase even with the normal range of urinary albumin [6]. Previous studies have demonstrated that overt clinical syndrome is preceded by a phase of microalbuminuria, so microalbuminuria can serve as an early predictor of the overt syndrome. Studies examining such prediction models had mixed results [7,8].

The screening of proteinuria is usually performed with the help of urine dipstick method, which is a semi-quantitative colorimetric test. It is quick but can give false positive results, so it should be followed by a quantitative test [9,10]. Twenty-four hour urinary protein estimation is a conventional method and is considered as the gold standard. But, it may be troublesome or sometimes inaccurate due to over or under urine collection [11].

The spot urinary protein to creatinine ratio (PC ratio) and the albumin to creatinine ratio (ACR) have been studied in patients with renal disease, diabetes, and preeclampsia to assess proteinuria. Albumin excretion is considered to reflect glomerular damage more accurately than total protein excretion, and albuminuria may be a marker of systemic endothelial cell dysfunction [12]. The majority of international organizations now recommend spot proteinuria tests in the assessment of suspected preeclampsia. ACR has been shown to be an accurate indicator of proteinuria in women with preeclampsia [13,14].

Albumin-creatinine ratio (ACR) and PC ratio are both measured in the random spot urine specimen. But ACR is more sensitive and rapid as compared to PC ratio. Thus, this test can be conducted in women attending antenatal clinics [15,16]. In this study, we have evaluated the correlation of mid trimester spot urinary ACR with various pregnancy complications.

## Materials and methods

This was a prospective cohort study conducted over a period of one year in the department of Obstetrics and Gynaecology, All India Institute of Medical Sciences (AIIMS), Bhopal. After institutional ethical approval and informed consent, 146 pregnant women between 14 to 28 gestational weeks were enrolled. But due to lost to follow up and missing data, only 130 pregnant women were included for analysis. Pregnant women with dipstick positive proteinuria, ongoing urinary tract infection (UTI), multiple pregnancy, chronic kidney disease (CKD), foetal anomaly, known medical diseases like hypertension and diabetes mellitus were excluded.

Demographic data like age, education, occupation, and socioeconomic status were recorded. Past or present medical history was noted. General examinations including blood pressure measurement and body mass index (BMI) were recorded. All routine antenatal investigations were done. Participants were asked to give spot mid-stream urine samples in a sterile container for quantitative measurement of albumin and creatinine. The albumin creatinine ratio was calculated as mcg (microgram) of albumin per milligram (mg) of creatinine. Urine routine microscopy was also performed. ACR was analysed by Beckman Coulter AU analyser (Beckman Coulter, Inc., Brea, CA). Participants were followed in each antenatal visit. During each antenatal visit, participants were screened for the development of gestational hypertension, pre-eclampsia, or any other complications. After delivery, neonatal outcome was evaluated in terms of birth weight, NICU admission, and APGAR scores.

Gestational diabetes mellitus (GDM) is defined as any degree of glucose intolerance with onset or first recognition during pregnancy [17]. At our institute, the diagnosis of GDM is based on the 75 gm glucose tolerance test. A two hour blood sugar value of > 7.8mmol/l is considered impaired glucose tolerance.

Gestational hypertension and preeclampsia were defined according to the National High Blood Pressure Education Programme Working Group 2000 (NHBPEP) and the American College of Obstetricians and Gynecologists (ACOG) [18]. Gestational hypertension was defined as a systolic blood pressure of 140 mm Hg or more or a diastolic blood pressure of 90 mm Hg or more, or both, on two occasions at least six hours apart after 20 weeks of gestation in a woman with previously normal blood pressure. Preeclampsia was defined as a disorder of pregnancy associated with new-onset hypertension, which occurs most often after 20 weeks of gestation with proteinuria and/or end organ damage. Although often accompanied by new-onset proteinuria, hypertension and other signs or symptoms of preeclampsia may present in some women in the absence of proteinuria [18].

Data entry and statistical analysis

The collected data were transformed into variables, coded, and entered in Microsoft Excel. Data were analyzed and statistically evaluated using SPSS software, version 25.0 (IBM Corp., Armonk, NY). Normal distribution of different parameters was tested by the Shapiro-Wilk normality test. Quantitative data was expressed in mean ± standard deviation or median with interquartile range and difference between mean of two groups was compared by mann Whiney U test while for more than two groups Kruskal Wallis H test was used. Sensitivity, specificity, positive predictive value (PPV), and negative predictive value (NPV) at different values of ACR for predicting pre-eclampsia/GDM/preterm labour were calculated from the receiver operating curve (ROC). P value less than 0.05 was considered statistically significant.

## Results

Prevalence of microalbuminuria in our study was 19.2% (range of microalbuminuria taken as 30-299 mg of albumin per gm of creatinine [1]). Mean maternal age was 26.35±4.76 years in our study. Out of total, 42.3% women hailed from lower-middle and 31.5% from upper lower socioeconomic status according to modified kuppuswamy scale (Table 1). In our study, mean urinary ACR was 19.07±12.94 mcg/mg and median urinary ACR (IQR) was 18 (9.43-25.25) mcg/mg. Out of total 130 women, 13 (10%) developed GDM, 16 (12.3%) had gestational hypertension during follow up. Seven (5.4%) women were diagnosed with preeclampsia and 26 (20%) women delivered preterm. None of the participant had eclampsia in our study. Sixty-four (49.2%) women delivered by lower segment caesarean section (LSCS). Twenty-two (16.9%) newborns needed NICU admission and 10 (7.7%) were born with low APGAR scores (Table 1).

**Table 1:** Demographic and clinical profile ACR: Albumin creatinine ratio; IQR: Interquartile range; GDM: Gestational diabetes mellitus; LSCS: Lower segment caesarean section; APGAR: Appearance, Pulse, Grimace, Activity, Respiration; NICU: Neonatal intensive care unit

Variable	No.	%
1. Mean age in years 26.35±4.76 years		
2. Socioeconomic status		
Upper	2	1.5
Upper middle	12	9.2
Lower middle	55	42.3
Upper lower	41	31.5
Lower	20	15.4
3. Gravida		
Primigravida	67	51.5
Multigravida	63	48.5
4. Mean BMI	20.75±2.47 kg/m^2^	
5. Mean urinary ACR	19.07±12.94	
6. Median urinary ACR (IQR)	18 (9.43-25.25)	
7. Maternal complication		
GDM	13	10.0
Gestational hypertension	16	12.3
Preeclampsia	7	5.4
Preterm labour	26	20.0
LSCS delivery	64	49.2
8. Foetal complication		
Low birth weight	37	28.5
Low APGAR	10	7.7
NICU admission	22	16.9

It was observed that urinary ACR level was significantly higher in women with maternal complication like GDM, gestational hypertension, preeclampsia, and preterm labour. Mean urinary ACR of patients who developed GDM was 28.12±13.60 as compared to 18.06±12.53 who did not show glucose intolerance during pregnancy (p =.005). (Table 2). Mean urinary ACR was higher in patients who had gestational hypertension and preeclampsia as compared to the unaffected women (p < .001 and p =.016, respectively). Also, mean urinary ACR of women who developed preeclampsia was higher (37.53±31.85) compared to women who developed gestational hypertension (27.40±9.71) (Table 2). It was found that spot urinary ACR was higher in women who delivered preterm (mean 25.74±19.32) as compared to the women delivered at term (mean 17.41±10.25). Although most of the preterm delivery were in women who developed preeclampsia, GDM or gestational hypertension.

**Table 2:** Association between urinary ACR levels and maternal outcome GDM: Gestational diabetes mellitus; ACR: Albumin creatinine ratio

Maternal outcome	Urinary ACR Mean ± SD	Range	P value
GDM			
Yes (n=13) 10%	28.12±13.60	7.61-55.0	0.005
No (n=117)	18.06±12.53	2.74-105.94	
Gestational hypertension			
Yes (n=16) 12.3%	27.40±9.71	8.56-45.0	<0.001
No (n=114)	17.90±12.94	2.74-105.94	
Preeclampsia			
Yes (n=7) 5.3%	37.53±31.85	10.30-105.94	0.016
No (n=123)	18.02±10.32	2.74-55.0	
Preterm labour			
Yes (n=26) 20%	25.74±19.32	5.0-105.94	0.013
No (n=104)	17.41±10.25	2.74-55.0	

In our study, it was found that women with higher urinary ACR delivered low birth weight babies (27.14±22.59 V/S 17.29±10.43) (p value = .058). Urinary ACR level was significantly higher in babies with low APGAR score (p value = 0.016) and who needed NICU admission (p value = 0.001) (Table 3).

**Table 3:** Association between urinary ACR and fetal outcome ACR: Albumin creatinine ratio; APGAR: Appearance, Pulse, Grimace, Activity, Respiration; NICU: Neonatal intensive care unit; NAD: Normal

Fetal outcome	Urinary ACR Mean ± SD	Range	P value
Birth weight			
<2.0 kg (n = 17)	27.14±22.59	5.54-105.94	0.058
2.0-2.49 kg (n = 20)	20.49±10.23	5-35	
≥2.5 kg (n = 93)	17.29±10.43	2.74-55.0	
APGAR score			
Low (n = 10)	26.50±10.16	5.54-45.0	0.016
NAD ( n = 120)	18.45±12.99	2.74-105.94	
NICU admission			
No (n = 108)	17.11±10.09	2.74-55.0	0.001
Yes (n = 22)	28.71±19.83	5.54-105.94	

**Figure 1:**
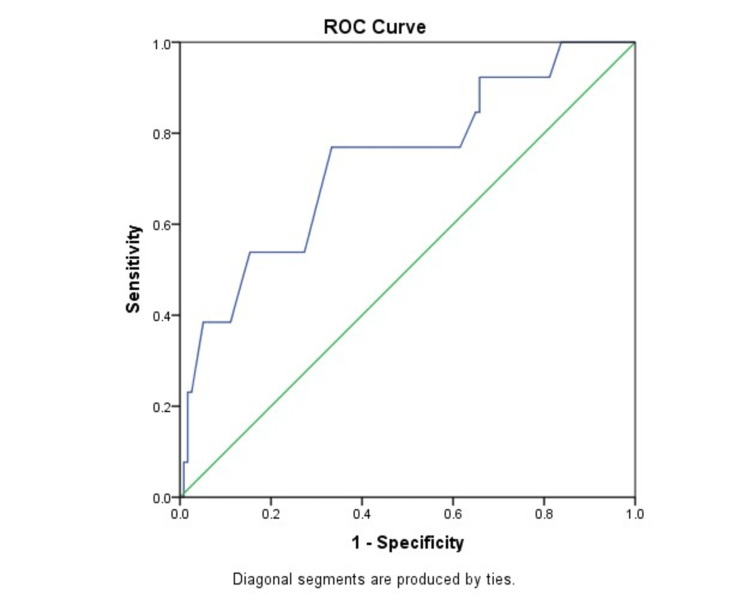
ROC curve using spot urinary ACR to predict GDM ROC: Receiver operating curve; GDM: Gestational diabetes mellitus; ACR: Albumin creatinine ratio

The sensitivity and specificity of spot urinary ACR to detect GDM was calculated from the ROC. The optimum spot urinary ACR to predict GDM was 22.32 mg/mmol, with a test sensitivity of 76.92%, specificity of 66.7%, PPV of 20.41%, and NPV of 96.3%. The area under the curve (AUC) was 0.73 (95% CI 0.58-0.89; P = 0.005) (Table 4, Figure 1).

**Table 4:** Diagnostic value of urinary ACR to predict different maternal complications ACR: Albumin creatinine ratio

	GDM	Preeclampsia	Preterm labour
Area under curve	0.73	0.77	0.65
95% CI	0.58-0.89	0.56-0.97	0.54-0.77
Cut off value	22.32	29	14.5
Sensitivity	76.92 (46.19-94.96)	71.43 (29.04-96.33)	76.92 (56.35-91.03)
Specificity	66.7 (57.36-75.11)	83.74 (76.01-89.78)	49.04 (39.1-59.03)

**Figure 2:**
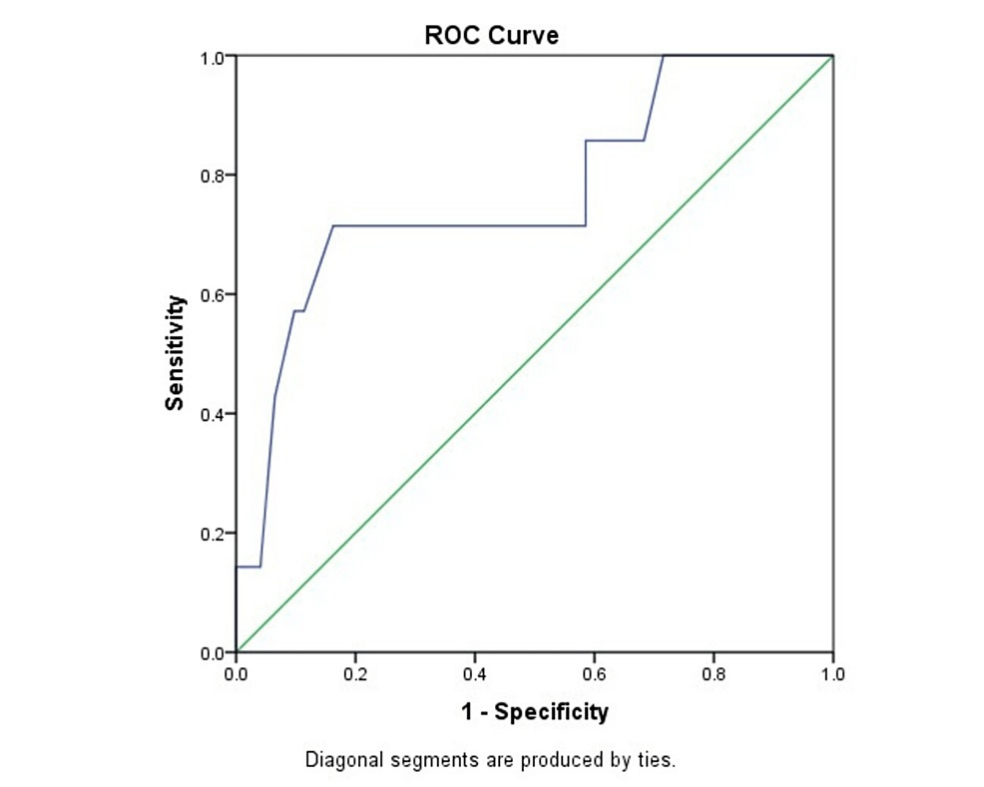
ROC curve using ACR to predict preeclampsia ROC: Receiver operating curve; ACR: Albumin creatinine ratio

The optimum spot urinary ACR to predict preeclampsia was 29 mg/mmol, which had a test sensitivity of 71.43%, specificity of 83.74%, PPV of 20.0%, and NPV of 98.1%. The area under the curve (AUC) was 0.77 (95% CI 0.56-0.97; P = 0.016) (Figure 2).

## Discussion

Persistent microalbuminuria is a marker of damage to the glomerular filtration capacity of the kidneys and may predict the development of preeclampsia and other complications during the course of pregnancy. Twenty-four hour urinary collection is considered the most accurate test for quantitatively estimating urinary protein, but it is time consuming [5]. Previous studies have shown fair correlation of urinary ACR with 24 hour urine protein and hence spot urinary ACR may replace 24-hour urinary protein estimation in future. Our present study evaluated the role of single spot urinary ACR as a screening test for various complications in pregnancy, as we hypothesize that microalbuminuria may precede the stage of gross proteinuria.

In our study, median urinary ACR (IQR) was 18 mcg/mg (9.43-25.25) as compared to the study conducted by Baweja et al. in which median urinary ACR was 28 mg/mmol (IQR 16-46 mg/mmol) [19]. According to that study, optimum ACR to predict preeclampsia was 35.5 mg/mmol, which is quite similar to our study in which the optimum ACR to predict preeclampsia was 29 mg/mmol. The incidence of preeclampsia in our study was 5.3%, comparable to 4-18% in developing countries [20]. In the present study, the mean spot urinary ACR value of women with preeclampsia was 37.53±31.85 mcg/mg compared to mean ACR of 18.02±10.32 mcg/mg of the unaffected women. Mishra et al. found that the mean spot urinary ACR of women who developed pre-eclampsia was significantly higher (51.95±18.78 mg/mmol) as compared to the normotensive women (19.26±7.99 mg/mmol) [21]. Baweja et al. [19] found the similar results in their study. 

Elia et al. found in a retrospective cohort study that urinary ACR is an independent prognostic factor for combined adverse maternal and neonatal outcomes. Their study established that a unit increase in log transformed ACR could increase the maternal adverse composite outcome by 30% and neonatal outcome by 10% [22].

Zen et al. found in their study that microalbuminuria detected in 1st trimester provides prognostic information regarding the risk of preeclampsia in women with pre-existing diabetes [23]. Bar et al. reported in their study that microalbuminuria detected in the early third trimester can predict the development of preeclampsia but is a poor predictor of intrauterine growth retardation (IUGR) and neonatal outcome [7]. Another study conducted by Poon et al. detected microalbuminuria in the first trimester in 55% of normal pregnancy and 75% of pregnancies which later develop preeclampsia. They also found that addition of urinary ACR to the prediction model does not improve the accuracy of the detection of preeclampsia [24].

## Conclusions

Our study found definite correlation of higher values of urinary ACR with the adverse pregnancy outcome. As this test has the ability to predict pregnancy complications, it can be incorporated with various screening models. Also, this test can replace the 24-hour urinary protein estimation in pregnant women owing to its accuracy and simplicity. Future studies with adequate sample sizes are needed to determine the exact cut-off values of urinary ACR, which can predict pregnancy complications with high sensitivity and specificity.
